# World Allergy Organization (WAO) Diagnosis and Rationale for Action against Cow's Milk Allergy (DRACMA) Guideline update – VII – Milk elimination and reintroduction in the diagnostic process of cow's milk allergy

**DOI:** 10.1016/j.waojou.2023.100785

**Published:** 2023-07-24

**Authors:** Rosan Meyer, Carina Venter, Antonio Bognanni, Hania Szajewska, Raanan Shamir, Anna Nowak-Wegrzyn, Alessandro Fiocchi, Yvan Vandenplas, WAO DRACMA Guideline Group

**Affiliations:** a Faculty Medicine, Imperial College London, Department Nutrition and Dietetics, Winchester University, UK and Faculty Medicine, KU Leuven, Belgium; b Children's Hospital Colorado, University of Colorado, Denver, CO, USA; c Department of Health Research Methods, Evidence & Impact, McMaster University, Hamilton, Ontario, Canada; d Evidence in Allergy Group; Department of Medicine and Health Research Methods, Evidence and Impact, McMaster University, Hamilton, Ontario, Canada; e Department of Paediatrics, The Medical University of Warsaw, Warsaw, Poland; f Institute of Gastroenterology, Nutrition and Liver Diseases, Schneider Children's Medical Center of Israel, Sackler Faculty of Medicine, Tel Aviv University, Israel; g Hassenfeld Children's Hospital, Department of Pediatrics, NYU Grossman School of Medicine, New York, NY, USA; h Allergy Unit - Area of Translational Research in Pediatric Specialities, Bambino Gesù Children's Hospital, Rome, Italy; i Vrije Universiteit Brussel, UZ Brussel, KidZ Health Castle, Brussels, Belgium

**Keywords:** Amino acid formula, Challenge test, Cow's milk allergy, Elimination diet, Extensive hydrolysate, Milk ladder, Oral challenge test, Rice hydrolysate, Soy formula

## Abstract

The diagnosis of cow's milk allergy (CMA) in infants and young children remains a challenge because many of the presenting symptoms are similar to those experienced in other diagnoses. Both over- and under-diagnosis occur frequently. Misdiagnosis carries allergic and nutritional risks, including acute reactions, growth faltering, micronutrient deficiencies and a diminished quality of life for infants and caregivers. An inappropriate diagnosis may also add a financial burden on families and on the healthcare system.

Elimination and reintroduction of cow's milk (CM) and its derivatives is essential for diagnosing CMA as well as inducing tolerance to CM. In non-IgE mediated CMA, the diagnostic elimination diet typically requires 2–4 weeks before reintroduction, while for IgE mediated allergy the time window may be shorter (1–2 weeks). An oral food challenge (OFC) under medical supervision remains the most reliable diagnostic method for IgE mediated and more severe types of non-IgE mediated CMA such as food protein induced enterocolitis syndrome (FPIES). Conversely, for other forms of non-IgE mediated CMA, reintroduction can be performed at home. The OFC cannot be replaced by the milk ladder after a diagnostic elimination diet. The duration of the therapeutic elimination diet, once a diagnosis was confirmed, can only be established through testing changes in sensitization status, OFCs or home reintroduction, which are directed by local protocols and services' availability. Prior non-evidence-based recommendations suggest that the first therapeutic elimination diet should last for at least 6 months or up to the age of 9–12 months, whichever is reached first. After a therapeutic elimination diet, a milk-ladder approach can be used for non-IgE mediated allergies to determine tolerance. Whilst some centers use the milk ladder also for IgE mediated allergies, there are concerns about the risk of having immediate-type reactions at home. Milk ladders have been adapted to local dietary habits, and typically start with small amounts of baked milk which then step up in the ladder to less heated and fermented foods, increasing the allergenicity.

This publication aims to narratively review the risks associated with under- and over-diagnosis of CMA, therefore stressing the necessity of an appropriate diagnosis and management.

## Introduction

Cow's milk allergy (CMA) is one of the most common and complex food allergies in infants and young children. It presents with many clinical manifestations overlapping with other conditions such as gastro-esophageal reflux and infantile colic. This results in misdiagnosis and improper management such as inappropriate prescription of medications and therapeutic formulas in non-breastfed infants.^1^

The prevalence of CMA during infancy was estimated to be 1.9% in a Finnish study, 2.16% in the Isle of Wight (United Kingdom), 2.22% in a study from Denmark, 2.24% in the Netherlands, and up to 4.9% according to Norwegian data.^2^ The British Society for Allergy and Clinical Immunology (BSACI) reported an estimated population prevalence of CMA between 2% and 3% during the first year of life.^3^ The incidence of CMA in exclusively breastfed infants is in the range of 0.4%–0.5% according to 2 trials^4^^,^^5^ but might be as high as 2.1% according to a cohort study of 824 exclusively breastfed infants.^6^ According to the data by Host et al, not more than 0.5% of the 2.2% children (meaning only 0.011% of all children) presented with a challenge proven IgE-mediated CMA whilst being exclusively breastfed.^3^^,^^7^

As part of the EuroPrevall study,^8^ 823 children were followed up until the age of 2 years in Hampshire (United Kingdom), yielding cumulative incidence estimates of 2.4% (1.4–3.5) for IgE-mediated CMA and 1.7% for non-IgE-mediated CMA.^9^ It remains unclear as to whether these differences reflect a different genetic background, a differential patient selection, or both. Other interfering factors may be confounding variables such as the difference in the gastrointestinal (GI) microbiome composition resulting from different modes of delivery (vaginal delivery *versus* caesarean section), feeding, pollution, and the administration of medication such as antibiotics and proton pump inhibitors (PPIs) early in life.^10^

IgE-mediated CMA is typically more easily recognized than non-IgE mediated allergy due to symptoms occurring relatively soon (typically within minutes to 1–2 h) after ingestion of cow's milk (CM), and the suspected diagnosis can be supported by elevated food-specific IgE levels or skin prick tests.^11^ Non-IgE CMA is more difficult to identify because the time interval between ingestion and symptoms ranges from a couple of hours to a few days, and the presentation might mimic pediatric functional gastrointestinal disorders (FGIDs), with symptoms like regurgitation, vomiting, diarrhoea, and constipation.^3^^,^^11^ For this reason, the Cow's Milk related Symptom Score (CoMiSS™) was developed to raise awareness about the fact that such non-specific symptoms may also be caused by (non-IgE) mediated allergy.^12^^,^^13^ Regardless, it is very important to accurately diagnose CMA to avoid the negative consequences either under- or over-diagnosis.

In this paper the WAO DRACMA Guideline panel, intends to give a narrative overview of previous literature on the topic and provide experts' opinions on the disease management. These are purely based on the personal expertise and judgment of the panel members. The methods underlying the guidelines’ development and the panel endorsed recommendations on CMA diagnosis and management will be illustrated and thoroughly described elsewhere.

### Diagnosis

One of the objectives of the WAO DRACMA Guideline is to propose a unified diagnostic process, adaptable to all needs of children with suspected CMA.^14^ (This topic will also be covered in DRACMA VIII and IX). The diagnostic process of CMA varies based on the health care system, as well as on the training and availability of health care professionals (HCPs). According to health economic data from the United Kingdom from 2010, it took an average of 2–6 visits to the general practitioner and 2.2 months from the presentation of typical CMA symptoms until CMA was considered.^15^ A “real world” study reporting about suspicion of CMA in 4 countries (Czech Republic, Germany, Belgium, and United Kingdom), found that the mean duration of symptoms before suspicion of CMA varied between 6.9 weeks (Belgium) to 24.0 weeks (United Kingdom), across a heterogeneous population with respect to age range (12.7 weeks–34.1 weeks).^16^

The magnitude of CMA under- and over-diagnosis as well as the nature of health consequences they translate into for patients will be further expanded in the WAO DRACMA Guidelines method paper and in the systematic review of diagnostic testing accuracy we are conducting to rigorously inform our decision-making process.

### Management of CMA

**Fig. 1:**
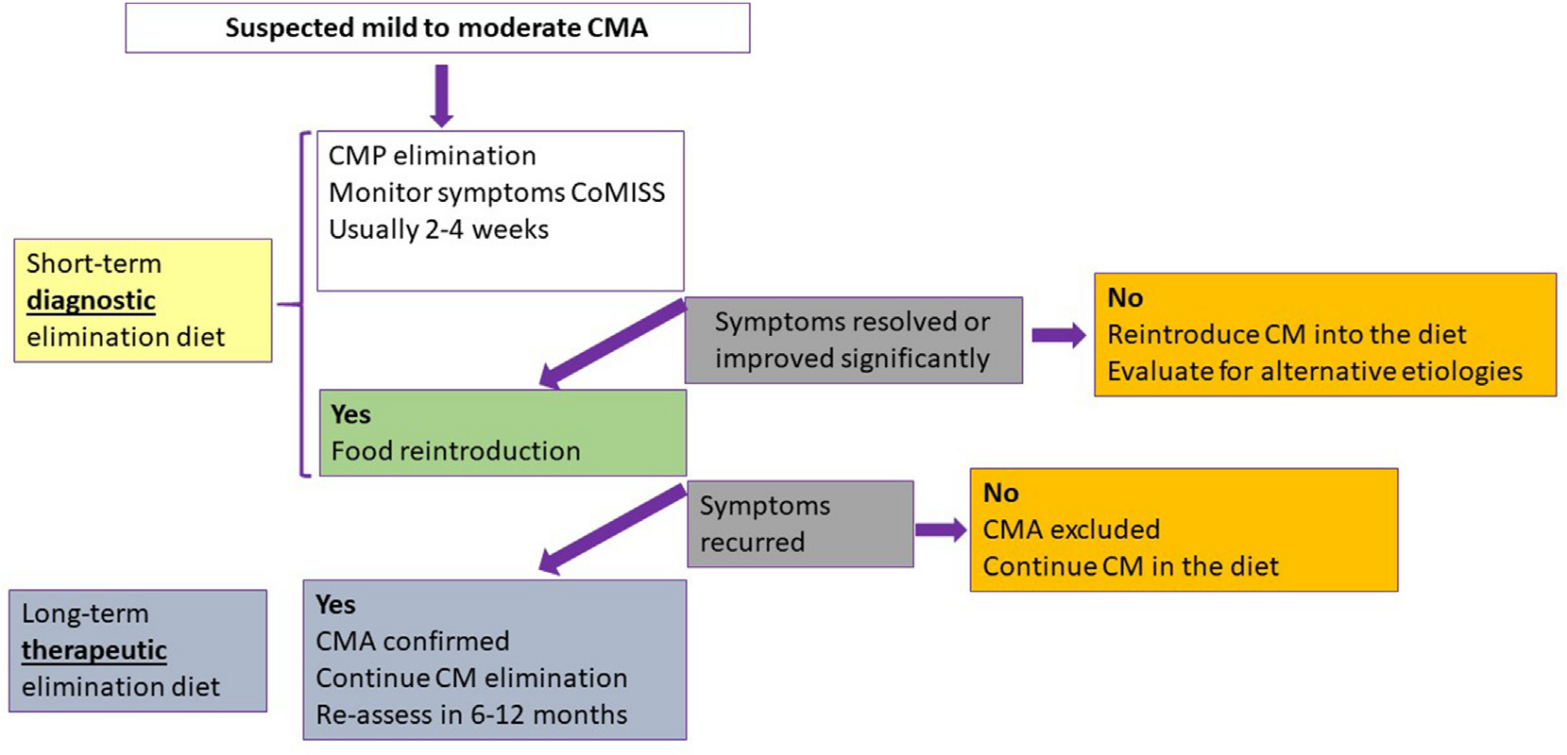
Recommended management in infants with cow's milk allergy. Schematic diagnostic and therapeutic elimination diet.

In the majority of infants with CMA, management consists of a three-step approach: i) a 2–4 week diagnostic elimination diet, ii) a food challenge for IgE mediated allergy and home reintroduction for non-IgE, and iii) a therapeutic elimination diet. An elimination diet means that all sources of CM should be eliminated from the diet of the child. If the symptomatic infant is exclusively breastfed, breastfeeding should be continued and the mother should be put on a 2–4 weeks CM-free diagnostic elimination diet (Fig. 1), after which CM should be reintroduced.

### Under-diagnosis

Because of the non-specificity of symptoms, children with CMA may not be labelled as such, and therefore not receive appropriate treatment. A delayed diagnosis has a detrimental impact on the child's health as allergen exposure results in allergic reaction and an underlying inflammatory status.^8^ Additionally, discomfort during food consumption can lead to feeding difficulties, further associated with a compromised diet.^17^ In symptomatic patients, medications, including corticosteroids, PPIs and dermatological products may be prescribed in preference to a CM elimination diet.^18^ The concerns about unwarranted administration of PPIs for “occult gastro-esophageal reflux” and “infant distress” have been highlighted in studies, and guidelines now advise using a CM elimination diet prior to considering PPIs.^19^^,^^20^ A diagnostic elimination diet should only be considered if CMA is truly suspected, ie, in the presence of other worrying signs like poor growth. Reflux in the absence of faltering growth does not warrant an elimination diet. Treatment of eosinophilic oesophagitis involves PPIs, swallowed topical steroid preparations, as well as dietary elimination.^21^ While food allergic children with asthma are at higher risk of anaphylaxis, CM is unlikely to be the cause of wheezing in asthma outside the context of anaphylaxis. Dermatitis may be attributed to atopic disease but may also be symptoms of CMA. Facial eczema was reported to be associated with development of CMA, but this does not mean causality.^22^ Sustained allergic inflammation resulting from allergic disease, as observed in CMA, atopic dermatitis and asthma cause impaired growth hormone release, malabsorption, increased nutrients' loss, and poor sleep quality and quantity.^23^ Inflammatory cytokines, particularly interleukin-6 produced by macrophages and tumour necrosis factor-α, play a central role in the development of allergic inflammation in atopic disorders. Studies in inflammatory bowel disease have established that these cytokines negatively impact longitudinal growth in infants.^21^^,^^24^ It follows that, when evaluating an infant with growth faltering, physicians should consider IgE mediated and non-IgE mediated CMA.^25^ Increased gut permeability may increase nutrients' demand and vitamin and mineral deficiencies, which are important cofactors for catch-up growth.^26^ Feeding difficulties may further reduce dietary intake and exacerbate the effect on growth.^17^

A delayed diagnosis of CMA is also associated with economic consequences, because of increased visits of HCPs and prescriptions of (ineffective) treatments.^1^ The burden for parents increases significantly in direct relation with the ongoing symptoms and the ineffective management.^27^ Ongoing symptoms will have a negative impact on the quality of life of the infants and the family.

**Figure:**
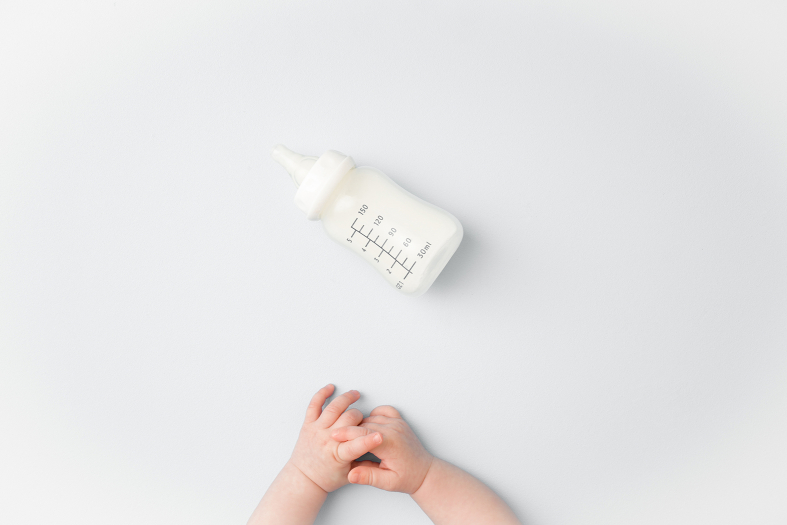

### Over-diagnosis

Over-diagnosis includes children who are treated for CMA, but who present with symptoms because of a different condition. These children are exposed to the harms of an unnecessary elimination diet. Over-diagnosis of CMA has been associated with several undesirable consequences, such as unwarranted elimination diets, and inappropriate dietary replacements for CM and its derivatives, which may, in turn, lead to feeding difficulties and insufficient nutrient intake.^17^

Non-IgE-mediated CMA presents with a multitude of symptoms, which are very common in infants and shared with other health conditions (Table 1).^28^ The over-diagnosis of CMA cannot therefore be attributable to unclear guidance alone, but also to ineffective implementation by healthcare providers.^29^ In support of this point, in the United Kingdom it was shown that the introduction of a simple and inexpensive training package led to prescription rate increase of hypoallergenic formulae by 63.2%, while alternative prescriptions decreased by 44.6% (P < 0.001), which translated into a reduction by 41.0% (P < 0.001) for all prescribed products used in primary care setting.^1^ Although the authors concluded that “this study shows promising results for prospective research on a national scale, including socio-economic impact and cost-effectiveness”,^1^ the outcome may as well suggest over-diagnosis of CMA.^30^ This observation illustrates that one of the major challenges in diagnosing (non-IgE) CMA versus FGIDs is the wide overlap between symptoms. It also highlights the fact that HCPs undergo pressure from parents “to do something”. Previous guidance has been criticized for promoting CMA over-diagnosis by labelling these symptoms as possible CMA-symptoms,^1^^,^^31^^,^^32^ even though the mandated dietary reintroductions, necessary for diagnostic confirmation are seldom performed (personal experience of the authors).

**Table 1:** Signs and symptoms associated with cow's milk allergy^b^

	IgE^a^	Non-IgE^a^
General	Anaphylaxis	Colic, irritability
		Failure to thrive
		Iron deficiency anaemia
Gastro-intestinal^c^	Regurgitation, VomitingDiarrhoea	Food refusal
		Dysphagia
		Regurgitation, vomiting^c^
		Diarrhoea^c^
		Constipation
		Anal fissures
		Perianal rash
		Blood loss
Respiratory c	Rhinitis and/or conjunctivitis	Rhinitis^d^
	Asthma	Wheezing^d^
	Mild dysphonia	Chronic cough^d^
Skin	Eczema (atopic dermatitis)	Eczema (atopic dermatitis)
	Acute urticaria^c^	
	Angio-oedema	
	Oral allergy syndrome	

a Patients may also present with mixed IgE and non-IgE symptoms.

b None of the symptoms is specific.

c Unrelated to infection.

d Upper and lower respiratory symptoms are sometimes attributed to non-IgE-CMPA but are not validated by blinded studies.

In a study by Munblit et al, 22% and 43% of parents reported vomiting and eczema in infants <12 months old, but CMA could be proven by oral food challenge (OFC) in only 0.7% of these.^33^ Very often, nutritional treatment, including extensive hydrolysates, is recommended and successful to alleviate symptoms of FGIDs in infants.^34^ Many of the therapeutic formulas contain a partial (whey) hydrolysate as protein source; however, since about 50% of the infants with CMA tolerate partial hydrolysate, symptom improvement upon receiving such supplements does not rule out CMA.^35^ Since there is no specific diagnostic test properly discriminating between non-IgE mediated CMA and FGIDs, and the recommended dietary approach may be effective in both conditions, this overlap will likely continue. Therefore, HCPs would be encouraged to properly follow dedicated guidance and apply a short-term diagnostic elimination diet followed by reintroduction/OFC, before embarking on a long-term therapeutic elimination diet.

Although CMA in exclusively breastfed infants is a rare condition, many breastfeeding mothers are put on unwarranted elimination diets contributing to premature and unnecessary discontinuation of breastfeeding, which might also have negative effects.^7^^,^^33^ Mothers can also independently, without medical advice, decide to start an (unnecessary) elimination diet.

In formula-fed infants, the economic aspect is of utmost importance because all therapeutic formulas suitable for CMA are much more expensive than standard infant formulas. From the nutritional point of view, it is safe to assume that if the volume of formula intake is adequate based on the infants’ age and weight, there is no safety concern since the formulas contain all required nutrients.^36^ Between the ages of 6 and 12 months, when complementary foods are introduced, intake of formulas may drop below 500 ml/day, making the addition of calcium and vitamin D through fortified foods or supplements essential. An important consideration in the unwarranted use of therapeutic formulas is that they have a different taste, due to the hydrolysis of protein and amino acids, which has been shown to have a potential long-term impact on taste preferences.^37^^,^^38^^,^^39^

Another negative consequence of an extended elimination diet appears at diversification, because of the limited possibilities, because many solid foods given to a baby between 6 and 12 months contain CM. Long lasting elimination diets, especially over the age of 1 year, can be associated with nutritional deficiencies, eating disorders and changes in taste preferences.^37^^,^^40^^,^^41^ In children older than 1 year with CMA who do not achieve tolerance, supplementation with calcium is recommended for the entire duration of the elimination diet. The intake of children with CMA differs significantly from a milk-consuming diet with respect to calcium, riboflavin, zinc, and niacin.^42^^,^^43^ Consuming a CM-elimination diet during infancy has persistent and long-term effects on eating habits and food preferences.^38^ A CM-elimination group (mean age 11.5 years) scored significantly higher on "slowness of eating" and on the combined "avoidant eating behavior" construct (p < 0.01).^38^ The number of avoided foods and symptoms were associated with higher levels of avoidant eating behavior (p < 0.05).^38^ The CMA group, who were instructed to avoid CM products in the first year of life, rated liking for several dairy foods (butter, cream, chocolate, full fat milk, and ice cream) significantly lower than the control group who consumed CM products during the first year of life (p < 0.05).^38^ Although there were no significant differences seen for any other category of food.^38^

Avoidance of a key food group such as CM compromises the intake of several nutrients, hampering the intake of sufficient energy, protein, vitamins, B, D, and A, minerals (especially calcium) and trace elements (eg, iron, zinc, and iodine).^44^^,^^45^ Since the absorption of calcium decreases from 30-40% to 10–15% when there is also vitamin D deficiency, calcium and vitamin D should often be supplemented in combination.^40^^,^^46^ The supplementary dose of elemental calcium can vary from 500 mg/day in infancy and toddlerhood to 1000 mg/day or more during adolescence^40^ depending on the national guidance and age of the child.^47^ Regarding vitamin D supplementation, patients at risk for vitamin D deficiency had a daily requirement of 400–1000 IU in the first years of life and 600–1000 IU from 1 to 18 years again depending on the national guidance and age of the child.^47^^,^^48^

Particular attention must be paid to protein-energy intake,^40^ as in a series of 130 children with a median age of 23.3 months and multiple allergies (mainly CM, soy, and egg) only 68.2% met the requirements for energy and 50.0% for protein.^44^ However, with appropriate nutrition counselling, children with food allergies reach the recommended levels of nutrients intake similarly to non-allergic children without a negative health impact.^36^^,^^41^^,^^49^

Carbohydrate and fat intakes may also be inadequate during an exclusion diet necessitating alternative sources in older children.^42^^,^^43^^,^^49^ In a cohort of 91 children with a mean age of 18.9 months (95% Confidence Interval 16.5–21.3), the plasma levels of linoleic, docosahexaenoic, and arachidonic acid lower compared to controls,^49^ suggesting that also these nutrients should be monitored while on elimination diets.

To prevent malnutrition in children with CMA, professional dietary advice is essential to ensure appropriate substitution of dairy products.^27^ Several studies have found improved nutrient intake in CMA children who receive dietary advice from a dietitian compared to those not receiving nutritional counselling.^45^^,^^50^

In the rare case CMA is suspected in a breastfed infant, the mother has to follow a strict CM elimination diet, necessitating often counselling by a dietitian and a specific management, for example receiving calcium and vitamin D supplementation.^7^

In clinical practice, during the diagnostic process of CMA, some children undergo the elimination of milk in all its forms, including the baked one, but also the elimination of all bovine proteins. This practice is not evidence-based and lacks a rational justification as less than 20% among those with IgE CMA are also allergic to beef.^51^ Specifically, the latter is associated with an allergy linked to bovine serum albumin, but the beef allergen is quite labile to temperature and digestion.^52^^,^^53^^,^^54^ So, cooking or baking beef destroys this allergen. It has been shown that practically all children allergic to beef do tolerate well-cooked beef meat.^55^ This is especially true if the meat is industrially prepared in a homogenized or freeze-dried form. As a consequence, it is not recommended to eliminate beef in CMA children who have not reacted to meat. In case meat has not been introduced, it can be introduced to all children allergic to milk in its thermally treated forms. This is particularly true in those already fed lamb which has shown an extensive cross-reactivity with beef.^56^

**Figure:**
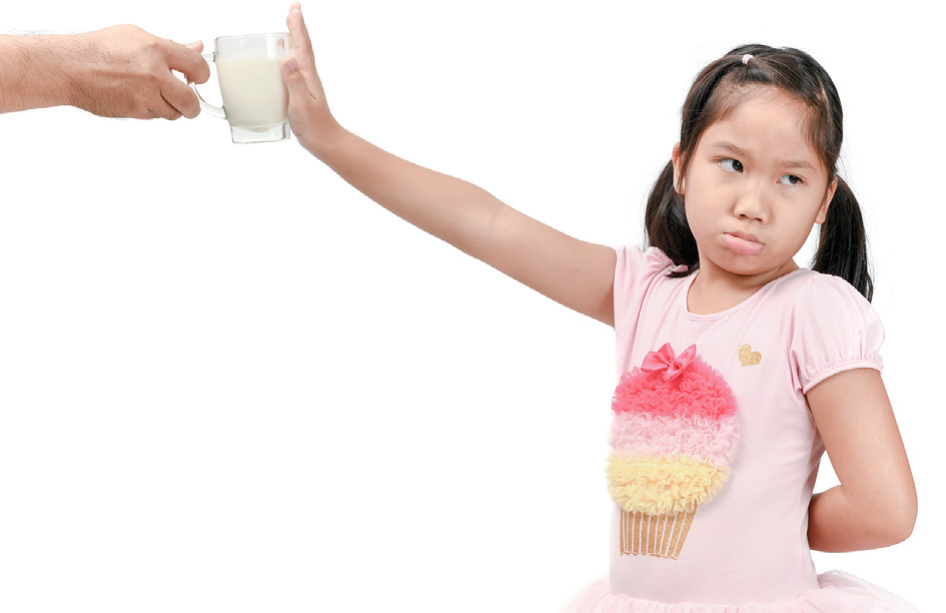

## Re-introduction to diagnose CMA

### Short-term diagnostic elimination diet

The standard procedure to diagnose CMA is an OFC after a 2–4 weeks elimination diet in non-IgE mediated allergy CMA. Although the dietary response in IgE mediated allergy may be faster, a similar duration of diagnostic elimination diet in IgE and non-IgE mediated allergy is usually advised.^57^ While in IgE mediated CMA, the OFC should be performed under medical supervision, the reintroduction in non-IgE allergy can be typically done at home. A confirmatory OFC is not recommended in patients with a history of anaphylaxis and in FPIES, unless there is uncertainty whether CM was the causative food.^57^^,^^58^ Although a double blind placebo controlled food challenge (DBPCFC) is the gold standard and best scientific approach, for practical and economic reasons an open milk challenge is recommended in infants. The DBPCFC is advised for scientific reasons and in case of a doubtful/inconclusive open challenge. It is important to continue a daily milk challenge with at least 200 ml/day for one week.^57^^,^^58^^,^^59^ The multi-step milk ladder is not a substitute for an oral food challenge (OFC) and is also not recommended for use to confirm a CMA. However, starting the reintroduction from a baked form of milk followed within 3–4 days by liquid milk is an accepted alternative approach for the caregivers who are apprehensive about retrying liquid milk.

### Therapeutic elimination diet

In case the OFC confirms the diagnosis of CMA, a therapeutic elimination diet for at least 6 months or up to the age of 9–12 months (whatever of both is reached first), is usually recommended.^57^ This is based on the observation that many infants with CMA become tolerant at this age, especially in case of non-IgE allergy.^8^ However, there is little to no scientific evidence in merit.^60^^,^^61^ No rigorous clinical studies were performed either to determine the best age to reintroduce CM or test the best duration of the therapeutic elimination diet.

A small study from Brazil reported that 80% infants with suspected CM proctocolitis tolerated CM by median age 6.3 months, suggesting that in case of food protein induced allergic proctocolitis (FPIAP), reintroduction attempts after age 6 months may be considered.^62^ This issue needs further attention since a large cohort study reported that CM FPIAP was associated with increased risk of developing IgE-CMA [adjusted odds ratio 5.4 (95% CI 1.4–20.8)] and raised concerns about the potential role of delayed introduction in IgE-CMA development across this vulnerable population.^63^^,^^64^ The presence or absence of other atopic manifestations should guide the health care practitioner (HCP) to start a diagnostic elimination diet or "watch and wait" for 1 month since the haematochezia disappears spontaneously in many breastfed infants.^65^ So, while formula fed infants presenting with FPIAP should be put on an elimination diet, if the infant is breastfed, mothers should not immediately be put on an elimination diet because the haematochezia often disappears spontaneously.^57^ If a diagnostic elimination diet is commenced, it is essential to reintroduce CM in the maternal diet and/or infant diet after 2–4 weeks, to avoid the unnecessary elimination.^57^

The rates of resolution for IgE-mediated CMA may be more delayed than for non-IgE CMA. An OFC to establish tolerance is necessary in most cases of IgE-mediated CMA and FPIES if the patient has been avoiding CM strictly.

If the reintroduction after the therapeutic elimination diet causes symptoms, there is consensus to continue the elimination diet for another 3 to 6 months, and then reintroduce CM again. However, there is no evidence supporting this recommendation as this was never studied. In IgE mediated CMA, if there are still detectable levels of specific IgE, reintroduction has to be performed under physician supervision in a medical setting, especially if the initial symptoms were severe. In case of mild to moderate non-IgE mediated CMA, the reintroduction can be performed starting with small volumes of milk after the initial therapeutic elimination diet, according to the milk ladder recommendations.^66^, ^67^, ^68^, ^69^

### Reintroduction after the diagnosis is established and a period of avoidance-therapeutic elimination diet

Ladders used for gradual reintroduction of food allergens into a food allergic individual's diet are increasingly being used internationally.^70^ The formal milk-ladder can be used for reintroduction in non-IgE mediated food allergy (FPIAP, and food protein induced enteropathy—FPE) and might be considered in carefully selected cases of IgE-mediated CMA and CM-FPIES to evaluate for tolerance after a period of therapeutic elimination diet (Table 2).^60^ In the milk ladder, cooked or baked milk is first introduced in small quantities, followed by higher doses of less thermally processed milk. The step-up is slow and gradual. There is not minimum or maximum time during which the ladder should be completed or how long each step should take as it is adjusted on individual patients' factors such as history, reactions, age, type of cow's allergy, and other clinical factors. Children may be tolerant to cooked or baked milk but still reacting to milk that was not heated. Milk in the form of processed yoghurt is also better tolerated.^61^ Standardization regarding the foods included in the ladder and medical considerations are required to practice patient-centered care, best assist patients and families, and ensure safety.

**Table 2:** Reintroduction of cow's milk proteins following a period of therapeutic elimination diet

	Milk ladder	Liquid milk
**IgE mediated CMA**		
Setting	• Usually under physician supervision in a medical setting • Selected cases might be considered for home reintroduction • younger than 3 years • without previous history of anaphylaxis or wheezingfrom any causes • skin prick test whealdiameter less than 8 mm for cow's milk	• Usually under physician supervision in a medical setting • At the physician discretion, home introduction might be considered for children who are known to tolerate milk in baked products and had mild symptoms to large amounts of liquid milk in the past
Pros	• Up to 70% of children who react to liquid milk, tolerate milk in a form of a baked product • High chance of success • Minimizes unnecessary milk elimination when access to food challenges is limited	• Straightforward • Short period • Easy to find products
Cons	• Prolonged process, more labour intense • Some forms of baked foods may not be appropriate for young infants • Children who react to baked milk tend to have more severe symptoms and higher risk of anaphylaxis	• More allergenic form of milk might induce uncomfortable symptoms • Children with feeding difficulties might refuse to try a new food in a medical setting under time constraint
**Non-IgE mediated CMA: FPIAP, FPE**		
Setting	• Usually done at home • Helpful when caregiver apprehensive/worried about reintroduction	• Can be done at home as symptoms are usually delayed, e/g., appear after few days • Typically lower GI tract involved: bloody stool, diarrhoea, discomfort
Pros	• Starting from less allergenic forms of foods at lower doses • Milder symptoms	• Straightforward • Short period • Easy to find products
Cons	• Prolonged process • More labour intense • Some forms of baked foods may not be appropriate for infants and young children	• More allergenic form of milk might induce uncomfortable symptoms
• **Non-IgE mediated CMA: FPIES**		
Setting	• Typically under physician supervision in a medical setting • Those with mild symptoms to large amounts of liquid milk might be considered for a very gradual home introduction	• Typically under physician supervision in a medical setting
Pros	• Some children with milk-FPIES might tolerate baked milk • More gradual, starting from lower doses of baked milk • Home setting usually more comfortable for infants and young children, more likely to try a new food in a familiar environment and unlimited time • Might induce milder symptoms from lower GI tract compared to violent vomiting in acute FPIES	• Clear indication of tolerance/reactivity • Short process (1 day) • Easy to find foods
Cons	• Unclear what % of FPIES patients is tolerant to baked milk • If tolerate baked milk, will need another trial for liquid milk • Risk of FPIES symptoms at home • Unclear if symptoms to baked milk would be milder than to liquid milk • Prolonged process, labor intense on the part of a caregiver • Some forms of baked foods may not be appropriate/well accepted by infants and young children • If introduction stopped for mild, non-specific GI symptoms, it may result in unnecessary prolonged elimination of milk from diet	• Larger dose might induce more violent vomiting • Usually intravenous access is required and can be difficult to secure • Child may refuse to eat the new food in an unfamiliar setting and under time constraint

Legend: GI, gastrointestinal; FPIAP, food protein-induced allergic proctocolitis syndrome; FPE, food protein-induced enteropathy; FPIES, food protein-induced enterocolitis syndrome.

Oral immune therapy (OIT) is limited to patients with IgE-mediated CMA and is the method of choice for preventing anaphylaxis and severe response to accidental exposure. OIT consists of daily ingestion of increasing doses of the allergen during the up-dosing phase, and ingestion of a constant dose during the maintenance phase based on specific tailored protocols.^70^ OIT in children with severe and persistent CMA deserves consideration, but currently this approach should be reserved for selected patients and restricted to specialized centers.

## Conclusion

The diagnosis of CMA remains challenging as both under- and over-diagnosis are associated with negative health outcomes and economic consequences. Based on current knowledge we would suggest diagnosing CMA by reintroducing CM 2–4 weeks after a diagnostic elimination diet at home in non-IgE mediated allergy and with a supervised formal OFC in IgE mediated CMA and FPIES. We would not suggest going through the extended milk ladder after a diagnostic elimination diet. If the re-introduction or OFC caused symptoms, a therapeutic elimination diet would be optimal for at least 6 months or up to the age of 9–12 months, whichever is reached first. In IgE mediated allergy, reintroduction to establish tolerance should be guided by the severity of symptoms and specific IgE and/or skin prick test. The appropriate timing for re-introduction of CM in the diet after the therapeutic elimination diet remains debatable as no randomized controlled trials (RCTs) were performed for this primary endpoint. After the age of 1 year, re-introduction with baked or cooked milk, according to a milk ladder adapted to local dietary habits, is preferable. There is a non-evidence based consensus to reintroduce CM every 3 to 6 months if symptoms persist after the initial therapeutic elimination diet during 6 months or up to the age of 1 year.

## Funding

This document was supported by the World Allergy Organization.

## Availability of materials and data

Not applicable.

## Author contributions

YV wrote the first draft of the manuscript RM, CV and YV worked equally on the next and final version(s); AB adapted and standardized the manuscript to WAO DRACMA Guideline standards; HS, RS, ANW, AF collaborated, reviewed, and agreed on the content.

## Ethics statement

Ethics approval was not required. The work of this review paper did not involve human subjects.

## Consent to publish

All authors agree to publication of this manuscript in World Allergy Organization Journal.

## Declaration of competing interest

RM has participated as a clinical investigator, and/or advisory board member, and/or consultant, and/or speaker for Abbott Nutrition, ELSE, Nestlé Nutrition Institute, Nestle Health Science, Nutricia/Danone and Mead Johnson.

CV reports grants from Reckitt Benckiser, grants from Food Allergy Research and Education, grants from National Peanut Board, during the conduct of the study; and personal fees from Reckitt Benckiser, Nestle Nutrition Institute, Danone, Abbott Nutrition, Else Nutrition, and Before Brands, outside the submitted work.

AB has no direct or indirect conflict to disclose.

HS academic-associated speaking engagements and/or received research funding from companies manufacturing infant formulae such as Arla, Danone, Hipp, Nestlé Health Science, Nestlé Nutrition Insitutie, Nutricia, Mead Johnson.

RS has participated as a clinical investigator, and/or advisory board member, and/or consultant, and/or speaker for Abbott, ELSE, Nestlé Nutrition Institute, Nestle Health Science, NGS, Nutricia, Soremartec and Ukko.

ANW receives research support from Alladapt Immunotherapeutics and Regeneron, speaking fees from Nestle, Danone, and Thermofisher; royalties from UpToDate; she serves as an Associate Editor for the Annals of Allergy, Asthma and Immunology, chair of the ABAI Board of Directors, director of the AAAAI Board, and the Chair of the Medical Advisory Board of the International FPIES Association.

AF has participated as a clinical investigator, and/or advisory board member, and/or consultant, and/or speaker for Abbott Nutrition, Danone, Soremartec, Novartis, Astrazeneca, Vertex, GSK, Sanofi, DVB, and Aimmune.

YV has participated as a clinical investigator, and/or advisory board member, and/or consultant, and/or speaker for Abbott Nutrition, Ausnutria, Biogaia, By Heart, CHR Hansen, Danone, ELSE Nutrition, Friesland Campina, Nestle Health Science, Nestle Nutrition Institute, Nutricia, Mead Johnson Nutrition, Pileje, Sanulac, United Pharmaceuticals (Novalac), Yakult, Wyeth.

## Members of the WAO DRACMA Guideline Group

Ignacio J. Ansotegui, MD, PhD (Department of Allergy & Immunology, Hospital Quironsalud Bizkaia, Erandio, Bilbao, Spain); Stefania Arasi, MD, PhD (Translational Research in Pediatric Specialities Area, Division of Allergy, Bambino Gesù Children’s Hospital, IRCCS, Rome, Italy); Amal H. Assa’ad, MD (Division of Allergy and Immunology, Cincinnati Children’s Hospital Medical Center, Cincinnati, OH, USA); Sami L. Bahna, MD, Dr PH (Allergy/Immunology Section, Louisiana State University Health Sciences Center, Shreveport, LA, USA); Roberto Berni Canani, MD, PhD (Department of Translational Medical Science, University of Naples Federico II, Naples, Italy); Antonio Bognanni, MD (Department of Health Research Methods, Evidence and Impact – HEI, McMaster University, Hamilton, ON, Canada); Martin Bozzola, MD (Department of Pediatrics, Pediatric Allergy/Immunology Section, British Hospital, Buenos Aires, Argentina); Jan Brozek, MD, PhD (Department of Medicine, Division of Clinical Immunology and Allergy, Department of Clinical Epidemiology & Biostatistics, McMaster University Health Sciences Centre, Hamilton, ON, Canada); Derek K. Chu, MD, PhD (Department of Medicine, Division of Clinical Immunology and Allergy; Department of Clinical Epidemiology & Biostatistics, McMaster University Health Sciences Centre, Hamilton, ON, Canada); Lamia Dahdah, MD (Translational Research in Pediatric Specialities Area, Division of Allergy, Bambino Gesù Children’s Hospital, IRCCS, Rome, Italy); Christophe Dupont, MD, PhD (Paris Descartes University, Pediatric Gastroenterology, Necker Hospital, Paris, Clinique Marcel Sembat, Boulogne-Billancourt, France); Piotr Dziechciarz, MD, PhD, Medical University of Warsaw, Warsaw, Poland; Motohiro Ebisawa, MD, PhD (Clinical Research Center for Allergy and Rheumatology, National Hospital Organization Sagamihara National Hospital, Kanagawa, Japan); Alessandro Fiocchi, MD (Translational Research in Pediatric Specialities Area, Division of Allergy, Bambino Gesù Children’s Hospital, IRCCS, Rome, Italy); Ramon Targino Firmino MD (Faculty of Medical Sciences of Campina Grande, UNIFACISA University Centre, Campina Grande, Paraiba, Brazil); Elena Galli, MD, PhD (Pediatric Allergy Unit, Research Center, San PietroFatebenefratelli Hospital, Rome, Italy); Rose Kamenwa, MD (Department of Pediatrics and Child Health, Aga Khan University Hospital, Nairobi, Kenya); Gideon Lack, MBBCh (Department of Women and Children’s Health/Peter Gorer Department of Immunobiology, School of Life Course Sciences, Faculty of Life Sciences & Medicine, King’s College London, UK; Evelina London Children’s Hospital, Guy’s and St Thomas’ Hospital NHS Foundation Trust, London, UK), Haiqi Li, MD (Pediatric Division Department of Primary Child Care, Children’s Hospital, Chongqing Medical University, Chongqing, China); Alberto Martelli, MD (Italian Society of Pediatric Allergy and Immunology, Milano, Italy); Anna H. Nowak-Wegrzyn, MD, PhD (Department of Pediatrics, New York University Langone Health, New York, NY, USA; Department of Pediatrics, Gastroenterology and Nutrition, Collegium Medicum, University of Warmia and Mazury, Olsztyn, Poland); Nikolaos G. Papadopoulos, MD, PhD (Allergy Unit, 2nd Pediatric Clinic, University of Athens, Athens, Greece; Division of Infection, Immunity & Respiratory Medicine, University of Manchester, UK); Ruby Pawankar, MD, PhD (Department of Pediatrics, Nippon Medical School, Bunkyo-Ku, Tokyo, Japan); Maria Said, RN (Allergy & Anaphylaxis Australia (A&AA), Castle Hills, New South Wales, Australia); Mario Sánchez-Borges MD (Department of Allergy and Clinical Immunology, Centro Médico-Docente La Trinidad Caracas, Venezuela) – posthumos; Holger J. Schünemann, MD, MSc, PhD (Department of Health Research Methods, Evidence and Impact (HEI), McMaster University, Hamilton, ON, Canada, and Cochrane Canada and McMaster GRADE Centre, Hamilton, ON, Canada); Raanan Shamir, MD, PhD (Institute of Gastroenterology, Nutrition and Liver Disease, Schneider Children’s Medical Center, Petach-Tikva, Israel; Sackler Faculty of Medicine, Tel-Aviv University, Tel-Aviv, Israel); Jonathan M. Spergel, MD, PhD (Division of Allergy and Immunology, Department of Pediatrics, The Children’s Hospital of Philadelphia, Perelman School of Medicine at University of Pennsylvania, Philadelphia, PA, USA), Hania Szajewska, MD (The Medical University of Warsaw – Department of Paediatrics, Warsaw, Poland); Luigi Terracciano, MD (Italian NHS and Italian Society of Social and Preventive Pediatrics, Milano, Italy); Yvan Vandenplas, MD, PhD (Department of Pediatrics, UZ Brussel, Vrije Universiteit Brussel, Brussels, Belgium); Carina Venter, PhD, RD (Section of Allergy & Immunology, University of Colorado Denver School of Medicine, Children’s Hospital Colorado, Aurora, CO, USA); Amena Warner, RN, SN (PG Dip) (Allergy UK, Planwell House, Sidcup, Kent, UK); Susan Waserman, MD, MSc (Division of Clinical Immunology and Allergy, Department of Medicine, McMaster University, Hamilton, ON, Canada); Gary W. K. Wong, MD (Department of Paediatrics, Faculty of Medicine, The Chinese University of Hong Kong, Hong Kong, China).

## Declarations, WAO DRACMA Guideline Group

Declaration of competing interest S Arasi, S Bahna, Bognanni, J Brozek, D Chu, L Dahdah, P Dziechciarz, E Galli, R Kamenwa, H Li, A Martelli, R Pawankar, H Schunemann, R Targino, L Terracciano, and A Warner have no conflicts to disclose. Relationships reported related to the submitted work: IJ Anstotegui – Abbott, Amgen, Astra Zeneca, Bayer, Bial, Faes Farma, Hikma, Menarini, Merck, Mundipharma, Roxall, Sanofi, Stallergenes, UCB. A Assa’ad – Aimmune Therapeutics, DBV Technologies, Astella, ABBVIE, Novartis, Sanofi, FARE, NIH and an intellectual property patent licensed to Hoth. R Berni Canani – Ch.Hansen, Danone, DVB, Humana, iHealth, Kraft Heinz, Mead Johnson, Nestlè, Novalac, Nutricia, Sanofi. M Bozzola – Danone C Dupont – Nestle Health Science, Nestle France, Nutricia, Novalac, Sodilac, Abbott, Danone, and stock ownership at DBV Technologies. M Ebisawa – DBV Technologies, Mylan, ARS Pharmaceuticals, Novartis. A Fiocchi – Abbott, Danone. G Lack – FARE, National Peanut Board (NPB), The Davis Foundation, Action Medical Research, UK Food Standards Agency, Medical Research Council, DBV Technologies, Mission Mighty Me, Novartis, Sanofi-Genyzme, Regeneron, ALK-Abello, Lurie Children’s Hospital. A Nowak-Wegrzyn – Nestle, Nutricia, Novartis, Gerber, Aimmune. N Papadopoulos – Novartis, Nutricia, HAL Allergy, Menarini/ Faes Farma, Sanofi, Mylan/Meda, Biomay, AstraZeneca, GSK, MSD, ASIT Biotech, Boehringer Ingelheim, Gerolymatos International SA, Capricare. M Said – Nestle, Nutricia, Abbott, Bayer for Anaphylaxis Australia. J Spergel – DBV Technologies, Regeneron, Sanofi, and Aimmune. H Szajewska – Ausnutria, Cargill, Danone, Else Nutrition, Hipp, Nestle, and Nestle Nutrition Institute. Y Vandenplas – Abbott Nutrition, Biogaia, Biocodex, By Heart, CHR Hansen, Danone, ELSE Nutrition, Friesland Campina, Hero, Hypocrata, Nestle Health Science, Nestle Nutrition Institute, Nutricia, Mead Johnson Nutrition, Orafti, Phacobel, Phathom Pharmaceuticals, Sari Husada, United Pharmaceuticals (Novalac), Wyeth, Yakult. C Venter – Reckitt Benckiser, Nestle Nutrition Institute, Danone, Abbott Nutrition, Else Nutrition, and Before Brands, DBV Technologies. S Waserman – Novartis-basic science work on peanut allergy, Aimmune-peanut OIT trial, Medical Advisor to Food Allergy Canada, and Pfizer, Bausch, Kaleoconsultant for epinephrine autoinjectors. GWK Wong – Nestle, Danone.
